# Malnutrition influences tumor necrotic factor-alpha (TNF-α) response among *Plasmodium falciparum* (*Pf*) malaria patients in Nigeria

**DOI:** 10.1186/1475-2875-13-S1-P3

**Published:** 2014-09-22

**Authors:** Olusola Ajibaye, Akiniyi Osuntoki, Bassey Orok, Bamidele Iwalokun, Nneka Egbuna, Yetunde Olukosi, Olugbenga Aina, Hilary Okoh, Chimere Agomo, Vera Enya, Oladayo Faneye, Samuel Akindele, Olutope Akinnibosun, Kolapo Oyebola, Sunday Adenekan, Albert Ebuehi

**Affiliations:** 1 Nigerian Institute of Medical Research, Lagos, Lagos State, Nigeria; 2 University of Lagos, Lagos, Lagos State, Nigeria; 3 University of Ibadan, Ibadan, Oyo State, Nigeria

## Background

In malaria endemic regions, malnutrition has also been reported to be a public health problem. Considering that the pattern of host cytokines-mediated innate immunity is critical in determining malaria outcomes-understanding the impact of malnutrition on innate immune response in *Plasmodium falciparum* (Pf)-infected patients may be helpful for malaria control. This study aims to determine nutritional status and evaluate the influence of malnutrition on the immune response of infected patients in Lagos, Nigeria.

## Methods

Volunteers (1,838) with a history of fever or axilliary temperature ≥37°C were screened microscopically for Pf in a cross-sectional study at Ijede General Hospital, Lagos, Nigeria. Body mass index (BMI) of patients was determined and used as a measure of nutritional status. A BMI of <18.5 kg/m^2^ was taken as an index of malnutrition while participants <20 years were further classified based on Z-scores (Z-Scores ≤-2) into stunted, wasted or underweight. TNF-α, interleukin-1β and interleukin-12 were determined by ELISA and haematological parameters (Full blood count) were measured using Beckman Coulter Closed Tube Automated Hematology System. Statistical analysis was done using SPSS Version 17. The study protocol was approved by NIMR Institutional Review Board.

## Results

A total of 364 patients comprising of 47% males and 53% females with a median age of 10 years were recruited for which average BMI was 18.19+6.10. Malaria prevalence was 20.85% and malnutrition rate 62.5%. In the <20 years group, 24 (16.7%) were stunted, 30 (20.8%) were underweight and 48 (33.3%) were wasted. TNF-α was associated with age, observed to be higher in <5 years (*P* = 0.001). Mean levels of TNF-α were significantly higher in malnourished patients *P* < 0.05.

## Conclusions

This study suggests that nutritional status modulates malaria outcomes and pattern of progression in all ages with an inference of high malnutrition rate in the studied population.

